# The Evolution and Expansion of Regional Disease Surveillance Networks and Their Role in Mitigating the Threat of Infectious Disease Outbreaks

**DOI:** 10.3402/ehtj.v6i0.19913

**Published:** 2013-01-25

**Authors:** Katherine C. Bond, Sarah B. Macfarlane, Charlanne Burke, Kumnuan Ungchusak, Suwit Wibulpolprasert

**Affiliations:** 1 Former Associate Director for Health, Rockefeller Foundation Southeast Asia and Africa Regional Offices, United States; 2 Department of Epidemiology and Biostatistics, School of Medicine, University of California San Francisco, United States; 3 Rockefeller Foundation, United States; 4 Department of Diseases Control, Ministry of Public Health, Thailand; 5 Office of Permanent Secretary, Ministry of Public Health, Thailand

**Keywords:** regional networks, disease surveillance, trust, pandemics, cross-border, SARS, International Health Regulations

## Abstract

We examine the emergence, development, and value of regional infectious disease surveillance networks that neighboring countries worldwide are organizing to control cross-border outbreaks at their source. The regional perspective represented in the paper is intended to serve as an instructive framework for others who decide to launch such networks as new technologies and emerging threats bring countries even closer together. Distinct from more formal networks in geographic regions designated by the World Health Organization (WHO), these networks usually involve groupings of fewer countries chosen by national governments to optimize surveillance efforts. Sometimes referred to as sub-regional, these “self-organizing” networks complement national and local government recognition with informal relationships across borders among epidemiologists, scientists, ministry officials, health workers, border officers, and community members. Their development over time reflects both incremental learning and growing connections among network actors; and changing disease patterns, with infectious disease threats shifting over time from local to regional to global levels. Not only has this regional disease surveillance network model expanded across the globe, it has also expanded from a mostly practitioner-based network model to one that covers training, capacity-building, and multidisciplinary research. Today, several of these networks are linked through Connecting Organizations for Regional Disease Surveillance (CORDS). We explore how regional disease surveillance networks add value to global disease detection and response by complementing other systems and efforts, by harnessing their power to achieve other goals such as health and human security, and by helping countries adapt to complex challenges via multi-sectoral solutions. We note that governmental commitment and trust among participating individuals are critical to the success of regional infectious disease surveillance networks.

## Introduction

The world has awakened to the threat of disease pandemics arising from growing global inter-connectedness. The rapid spread of SARS from Hong Kong to Toronto in 2003 demonstrated the speed with which highly pathogenic epidemics can move across the world, necessitating surveillance and control approaches that cut across national boundaries (1). Governmental and international agencies are building and strengthening infectious disease surveillance at all levels, from the national to international, to facilitate earlier detection and communication of disease outbreaks on a global scale (2–7). Here, we tell the story of surveillance networks that neighboring countries worldwide are organizing to control outbreaks at their source, across national borders. Distinct from more formal networks in geographic regions designated by the World Health Organization (WHO), these networks usually involve groupings of fewer countries chosen by national governments to optimize surveillance efforts. Sometimes referred to as sub-regional, these “self-organizing” disease surveillance networks complement national and local government recognition with trust-based relationships between practitioners across borders. Governments, public health authorities, international organizations, academia, foundations, and non-governmental organizations mobilize technical and financial support for these networks. Some of these regional networks existed before the sudden outbreak of SARS (2003). We describe their emergence, development, and value among the many other parallel efforts to protect populations against the global spread of infectious disease, with a focus on three of the earlier emerging networks: the Pacific Public Health Surveillance Network (PPHSN) (1996), the Mekong Basin Disease Surveillance (MBDS) network (1999), and the East African Integrated Disease Surveillance Network (EAIDSNet) (2000). The regional perspective represented in the paper is intended to serve as an instructive framework for others who decide to launch or join existing regional networks as new technologies and emerging threats bring countries even closer together.

## The Rise of Self-Organizing Regional Disease Surveillance Networks

Regional networks have increased in response to changing global disease dynamics. Zoonotic diseases account for 70 percent of emerging infectious diseases, and the global health and economic impacts of SARS, H5N1 and H1N1 have turned the world's attention to global pandemic threats. Common drivers include increased cross-border trade, mobility and migration of humans and animals, livestock productions systems, population density, viral adaptation and ecological shifts as a result of climate change (8).

While some of the more recently emerging regional disease surveillance networks are research-focused, the earlier networks were practitioner-based and aimed to bring together epidemiologists responsible for surveillance. The earliest example is the Organisations de Coordination et de Cooperation pour la lutte contre les Grandes Endemies (OCCGE), established in West Africa in the early 1960s. In 1987, the OCCGE merged with the West African Health Community (WAHC) to form the West African Health Organisation (WAHO) (9).

Several other regional disease surveillance networks arose in the mid-1990s. The Pacific Public Health Surveillance Network (PPHSN) formed in 1996 as a voluntary network to coordinate efforts to control infectious disease in 22 Pacific Island countries and territories. PPHSN met a need among countries and island territories in the region to streamline their disease reporting and response. The network operates under the auspices of the WHO Western Pacific Regional Office and the Secretariat of the Pacific Community (SPC).

In 1999, technical representatives of the six Mekong countries (Cambodia, China, Lao PDR, Myanmar, Thailand and Vietnam) recommended to their governments formation of the Mekong Basin Disease Surveillance (MBDS) network. This small number of countries bridging the much larger WHO Southeast Asia and Western Pacific regional offices, and also forming a subset of the Association of Southeast Asian Nations (ASEAN), decided that coordination through MBDS would enable them to address similar epidemiological profiles across their multiple shared borders.

The following year, in 2000, representatives from ministries of health and academic institutions in Kenya, Tanzania and Uganda formed the East African Integrated Disease Surveillance Network (EAIDSNet). This network supported the WHO African regional office's policy of integrating disease surveillance systems (10). Anticipating and supporting the development of a health desk within the re-emerging East African Community, countries familiar with working together wanted to prepare common procedures for combating disease threats such as Ebola or, more commonly, cholera (11). Rwanda and Burundi joined EAIDSNet in 2007.

## Phases of Network Development

We characterize the evolution of PPHSN, MBDS and EAIDSNet in three overlapping phases (1996–2007; 2003–2009; and 2006-present). These phases reflect both incremental learning and growing connections among network actors and changing disease patterns, with infectious disease threats shifting over time from local to regional to global levels. The occurrence of SARS during the first phase, H5N1 during the second phase, and H1N1 during the third phase reinforced the roles of these networks and strengthened the resolve of members to prepare joint plans to combat such threats.

### Phase 1: Training and connecting people to contain local epidemics (1996 to 2007)

During the early phases of network formation, populations struggled with diseases of primarily local concern. Priorities for disease reporting reflected the epidemiology of the time: diarrheal diseases like cholera; malaria; pneumonia; typhoid; hemorrhagic fevers like dengue; and HIV/AIDS and tuberculosis.

As the first step in establishing these networks, their members made and formalized connections among technical experts across the ministries of health and other public health institutions in the countries and agreed on strategies. The PPHSN formed a coordinating body, with representatives of the countries and territories and of allied bodies, including as permanent members WHO, the Secretariat of the Pacific Community, and Fiji School of Medicine (12). MBDS established a secretariat in Thailand, with coordinating and executive committees; and outlined a collective vision for governance and outputs in a memorandum of understanding signed by ministers of health for all six countries. EAIDSNet operated a temporary secretariat with a coordinating committee based in the Tanzanian National Medical Research Institute until it formally incorporated into the East African Community (EAC) in 2004.

Also during this first phase, the networks identified priority diseases and clarified disease definitions; harmonized reporting tools; prepared joint outbreak investigations; and disseminated information through network publications. PPHSN set up PACNet as an early warning system for disease outbreaks (Text Box 1); MBDS and EAIDSNet shared only limited surveillance information and undertook minimum collaborative response activities during this early phase of network formation.

***Text Box 1*. PACNET**

The PPHSN set up PacNet in 1997 to share timely information on disease outbreaks in order to ensure appropriate action was taken in response to public health threats (12). PacNet demonstrated early success by providing early warnings of dengue, measles, and influenza that led to preventive measures taken across the region. Its early success served as a building block for prioritizing regional surveillance of outbreaks of emerging and re-emerging diseases. Subsequently, all major outbreaks that posed threats to the region were notified and monitored through PacNet. These included SARS; dengue in Tong, New Caledonia, and Wallis and Futuna; measles in the Marshall Islands and Guam; rubella in Samoa, Niue and Tokelau; and influenza in New Caledonia.

All three networks focused on training health workers in field epidemiology, as the epidemiology workforces in some countries were not fully developed. For example, between 2001 and 2007, under the leadership of the Field Epidemiology Training Program in Thailand, MBDS trained 45 medical doctors in field epidemiology, disease surveillance, and response. Many of the individuals who completed the course later went on to lead or play significant roles in their respective ministries/countries; the relationships established during the course fostered the growth of informal, inter-governmental networks and future collaboration.

### Phase 2: Enhancing cross-border and national surveillance systems to address regional threats (2003 to 2009)

As HIV/AIDS spread increasingly in border areas and new zoonotic infectious diseases such as SARS emerged, a growing number of countries expressed heightened commitment to comply with the revised International Health Regulations (13). The networks used the communications infrastructure and the institutional agreements, arrangements, and definitions established early on to enhance cross-border disease surveillance and control projects. Both MBDS and EAIDSNet accelerated their efforts to strengthen district health management teams at border districts and to collaborate in the development of training programs for enhanced surveillance and control efforts.

At the same time, individual networks successfully integrated local, national and regional level health officials. For example, MBDS utilized existing bilateral and multilateral agreements between governments in the region to expand its cross-border initiative from four border sites in 2007 to 24 sites in 2010, effectively covering almost all key border crossings in the region. These combined capacities and growing trusted relationships further enabled collaboration in preparing for and responding to H5N1, dengue outbreaks, and natural disasters such as Cyclone Nargis that hit Myanmar in 2008 (14).

Also during phase 2, network activities moved beyond the health sector to include other sectors at border areas, including customs and immigration (Text Box 2). Multi-sectoral reporting teams highlighted that the spread of HIV/AIDS was significantly higher in border areas of the Mekong region than in other areas; and that East African countries were facing burgeoning cross-border epidemics, including HIV/AIDS but also Rift Valley Fever and other zoonoses (Text Box 3).

***Text Box 2*. Cross-border activities in the Mekong Basin region**

Building on good bilateral agreements among governments of the six countries and led by Lao PDR, which shares borders with all other MBDS countries, during phase 2 of its development MBDS created several multi-sectoral border response teams comprised of health, customs, immigration, and border officials. Through the cross-border sharing of human resources and expertise, the teams participated in a number of joint outbreak investigations. These included a joint dengue fever investigation between the Lao and Thai provincial sites, enabling officials to effectively stamp out the cross-border outbreak; a joint typhoid investigation between the Lao and Vietnam provincial sites; and a joint avian influenza investigation of cases in humans, triggered by the discovery of an infected Lao citizen in Thailand (15). Also during phase 2, MBDS partnered with Mahidol University, Thailand, to train border health officials in geographic information system (GIS) and other analytic techniques and in the social, political, and economic aspects of border health. This training enhanced skills in research, outbreak investigation, and communication; and established friendships and trust among officers from adjacent cross-border provinces.

***Text Box 3*. EAIDSNet: focusing on the animalhuman health interface**

During phase 2, EAIDSNet responded collectively to numerous outbreaks of cross-border significance, including Rift Valley Fever (2007), Marburg (2007), and wild poliovirus (2006, 2009, 2010). The outbreaks prompted a greater focus on the animal-human health interface and the need to develop integrated surveillance strategies. Thus, with a focus on One Health, the network conducted field simulation exercises at the Kenya-Uganda border to test national avian influenza preparedness and response plans; and conducted a review of information and communication technology (ICT) capabilities and developed and piloted a web portal linking existing human and animal disease surveillance reporting systems across facilities in border districts (11).

## Phase 3: Strengthening preparedness for pandemics and other public health threats of regional and global scale (2006 to present)

Growing global concern about the threat of H5N1 pandemic and other emerging infectious diseases prompted previously independent regional networks to start sharing experiences and learning from each other. In 2007, representatives from MBDS, EAIDSNet, and other regional networks from Southeast Asia, East Africa, and the Middle East met in Bellagio, Italy to discuss possibilities for collaboration (16). The Bellagio meeting was followed by a series of exchange visits during which the networks shared approaches to pandemic preparedness across regions and jointly piloted new information and communications technology (ICT) tools for communicating about disease outbreaks. Network members from MBDS report that these cooperative efforts helped strengthen their pandemic preparedness, citing improved national surveillance efforts and cross-border communication during the H1N1 outbreaks of 2009 (17). The Bellagio meeting also led to creation of Connecting Organizations for Regional Disease Surveillance (CORDS) (Text Box 4).

***Text Box 4*. CORDS**

In 2007, the Rockefeller Foundation (RF) supported the Nuclear Threat Initiative (NTI) to convene a Bellagio meeting of regional surveillance networks from across the globe to initiate a dialogue about how to harness lessons learned, emerging technologies, and nascent support. Participants from many networks recognized the value in sharing approaches and strategies, while donors and other development partners recognized the opportunity to reduce fragmentation and increase efficiencies in the global surveillance space. Subsequently, RF, NTI, and existing regional surveillance networks created a community of practice, “Connecting Organizations for Regional Disease Surveillance” (CORDS) (21, 22). Among its first activities, the community formulated a steering group comprised of key regional network representatives to define a learning agenda. More recently, CORDS registered as a legal, non-profit international organization in Lyon, France, in 2012. CORDS will convene the 1st Global Conference on Regional Disease Surveillance Networks at the Prince Mahidol Award Conference in 2013. Through these and other activities, CORDS is strengthening regional disease surveillance networks and global capacity for early detection and mitigation of pandemic threats.

## Expansion of Regional Networks

Not only has the regional disease surveillance network model expanded across the globe, it has also expanded from a mostly practitioner-based network model to one that covers training, capacity-building, and multidisciplinary research. This section describes more recently formed efforts.

### Practitioner Networks

The governments of Albania, Bosnia and Herzegovina, Bulgaria, Croatia, Moldova, Montenegro, Romania, and the former Yugoslav Republic of Macedonia established the Southeastern European Health Network (SEEHN) in 2001. SEEHN's goal is to foster cross-border collaboration to align national practices with European Union standards and requirements. Key activities include joint preparation of influenza pandemic preparedness plans at both national and regional levels and the introduction of molecular techniques into influenza surveillance laboratories within the region (18).

In 2003 representatives of the ministries of health of Israel, Jordan and the Palestinian Authority established the Middle East Consortium on Infectious Disease Surveillance (MECIDS) (2). MECIDS began as a set of informal working relationships among technical staff for monitoring food-borne outbreaks. Like the earlier networks, they began by harmonizing reporting methodologies; conducting joint training; sharing data and analysis; facilitating cross-border communication; and responding to cross-border outbreaks. Eventually, the network expanded its focus beyond food-borne outbreaks. MECIDS employed preparedness planning exercises in response to an outbreak of H5N1 in 2006; and conducted a series of national pandemic influenza table-top simulation exercises between 2007–2008, culminating in a regional exercise in 2008. These efforts contributed significantly to the region's response to the 2009 H1N1 outbreaks, putting into place many of the plans that emerged from the exercises (19, 20).

The Association of 10 Southeast Asian Nations (ASEAN) Plus Three (China, Japan, Korea) Field Epidemiology Training Network (ASEAN+3 FETN) was formalized in 2011. A permanent coordinating office was set up in Thailand, with support from the Thai Ministry of Public Health; and a steering committee formed, with the chair rotating annually. The network holds biannual steering committee meetings, videoconferences every three months, and special videoconferences during outbreaks or other emergencies (e.g., during a severe outbreak of hand, foot, and mouth disease (HMD) in China, Japan, Vietnam and Thailand in 2012; and when Thailand faced unprecedented nationwide flooding in 2011). A 2012 videoconference was called to discuss mysterious deaths among villagers in the central province of Vietnam that were attributed to a disease called inflammatory palmoplantar hyperkeratosis (IPPH) syndrome (23). ASEAN+3 FETN builds on the social capital created by MBDS and complements MBDS efforts toward strengthening field epidemiology capacity in the region.

### Research Networks

The Asia Partnership on Emerging Infectious Disease Research (APEIR) began in 2006, initially with a focus on avian influenza research (24). APEIR covers Cambodia, China, Indonesia, Laos, Thailand and Vietnam. Building on the MBDS model of regional cooperation and including many of the same individual members and country representatives, APEIR also brought in the agriculture, education and sciences sectors from academia to promote interdisciplinary research collaboration. The network comprises 30 partner institutions that collaborate on research and policy advocacy. Distinct from the more operational surveillance networks, APEIR's chronology was to begin with identification of research priorities from individual countries as well as inter-country issues. In its second phase, a coordinating office was established, hosted by the Health Systems Research Institute of Thailand, to coordinate funding support for research projects. The network currently focuses on dissemination and new collaborations in research. Research topics include ecological drivers of emerging infectious diseases such as migratory bird pathways and backyard poultry systems; and socioeconomic impact studies and policy analysis. The network also focuses on strengthening research capacity among its members.

The Southern African Centre for Infectious Disease Surveillance (SACIDS) was formalized in 2008 as a consortium of academic and research institutions with a One Health focus (25). It aims to strengthen capacity to detect, identify and monitor infectious diseases of humans, animals, and plants and their interactions in order to better manage the risks posed by them. SACIDS bridges the ministries of human health, livestock and wildlife in the 14 Southern African Development Community (SADC) countries and brings together infectious disease researchers from multiple sectors. Sokoine University of Agriculture, Tanzania, serves as a formal institutional base for the network. The focus of SACIDS is on academic training and research capacity development. The network has also been working with EAIDSNet to pilot an android driven mobile telephone system for rapid cross-border communication of animal-human health surveillance information.

## Value of Regional Networks

Infectious disease patterns reflect dynamic systems of global interconnectedness. Networks are not only the means through which rapid diffusion of disease can spread, but they can also become a critical alternative to formalized institutional responses to outbreaks. This is especially true for issues of transnational concern where formal governance structures are inadequate (26). Here, we explore how regional disease surveillance networks add value to global disease detection and response and the challenges they face.

*1. Complementing global, other regional, and country disease surveillance systems*. The WHO leads the global response to disease outbreaks, not just from its headquarters in Geneva but also at the regional and country levels, taking advantage of expertise available through partner institutions. In 2000, recognizing that comprehensive surveillance depends on many different players and networks, WHO created the Global Outbreak Alert and Response Network (GOARN) which serves as a network of networks “to link this expertise and skill to keep the international community constantly alert to the threat of outbreaks and ready to respond” (27). More recently, the International Health Regulations (IHR) mandate official reporting of certain types of disease outbreaks to WHO. Complementing WHO efforts are a range of global internet-based networks and electronic search engines such as the Public Health Agency of Canada's Global Public Health Intelligence Network (GPHIN) (28) and ProMed Mail (29). These efforts aim to facilitate surveillance on a global scale, while offering new opportunities for information sharing and access. Global disease surveillance still faces challenges in reporting due in part from the lack of national disease surveillance capacity in lower and middle-income countries; limited diagnostics capabilities; and disincentives to reporting due to harsh economic consequences (30, 31). Regional disease surveillance networks have addressed some of these limitations, and have helped to cross the geographic and topical boundaries of the largely vertical networks under WHO leadership. For example, MBDS has helped to connect a small number of countries that share borders but reside within two separate WHO regions.

*2. Harnessing network power*. The regional networks described here and throughout this issue of *Emerging Health Threats* have gained interest among some scholars for their contributions to global health security, particularly for their role in implementing the International Health Regulations (2005) (2, 32). However, other scholars have critiqued the health security motive as driven by trade interests and fears of bio-terrorism. Critics argue that vertical disease surveillance networks sometimes unfairly challenge low-income countries to upgrade their surveillance capacity for the benefit of more developed countries; and that efforts to harmonize definitions, detection and reporting benefit nations with more advanced surveillance systems and developed economies more than they benefit less advantaged nations (4, 6, 33, 34). If many low and middle-income countries lack access to the tools and knowledge to participate effectively, the networks are rendered less significant to the global response and less relevant to their own needs. Indeed, in coining the term “network power,” Grewal (35) suggested that globalization implicitly benefits powerful nations and groups through the establishment of dominant “standards” adopted through networks.

But network power can work to the advantage of low and middle-income countries. We posit that investing in regional disease surveillance networks strengthens national health systems and regional and global cooperation, thereby promoting health security everywhere (36). Regional disease surveillance networks prioritize building trust-based relationships that enable informal reporting and the rapid sharing of sensitive information; and enabling cross-border collaboration and the strengthening of technical capacity to detect and respond to infectious diseases in peripheral border areas with marginalized populations. When organizing their networks, nations and individuals make decisions based on local needs and priorities. In sum, regional networks strengthen *social and intellectual capital, capacity, and connectedness*.

Network power of this type is maximized when multiple mechanisms in the region are well coordinated. For example, in the Mekong region there are many mechanisms in addition to MBDS that can enhance disease surveillance, including WHO, ASEAN, the Asian Development Bank Mekong Project, and Aryawadee-Chaophraya-Mekong Economic Cooperation Strategies (ACMECS). The coordinating office or steering committee of each regional network needs to recognize and work with these other mechanisms to avoid duplication and to utilize the strengths of each mechanism. For example, with WHO or ACMECS support, MBDS was able to involve Myanmar in regional activities when funding for Myanmar was embargoed due to political sanctions by western governments.

*3. Adapting to complex challenges*. As network scientist Albert-Laszlo Barabasi pointed out, “The truly important role networks play is in helping existing organizations adapt to rapidly changing conditions. The very concept of network implies a multidimensional approach” (37). The rapidly changing conditions associated with infectious disease spread require multinational, multi-sectoral, multi-disciplinary solutions. In part because of their local reach, regional disease surveillance networks can contribute to these solutions by engaging other institutions and sectors in their efforts to establish multi-sectoral cross-border outbreak response teams or multi-disciplinary research teams.

*4. Making networks work*. Multi-country networks work when principles of sovereignty are maintained, when trust and confidence are established, and when technical professionals can freely deliberate and make collective decisions (38, 39). Further, the networks featured in this supplement illustrate other essential features as outlined by Anklam (40). Each regional network began with voluntary, not mandatory, participation; involvement is based on expertise, not by formal position, with expertise becoming available to the network as needed; and network members have a sense of belonging that fosters trust and cooperation. In sum, the networks featured here have cultivated a growing capacity to detect and curtail global and regional threats through local action and collaboration.

## Future challenges

The regional networks described in this paper have been supported by governmental commitments of personnel and expertise and essential catalytic funding by philanthropic partners. For example, the Rockefeller Foundation, with its ability to work flexibly with a range of institutions – from governments to academic institutes to non-governmental partners – awarded a combination of grants to regional networks in support of research; training; information and technology innovation; policy-making among government, academia, and other sectors; and travel and communications to enable members of different networks to connect with each other. The Foundation approach required a delicate balance to ensure that complementary interests among all partners were met as the networks worked toward achieving their respective common goals. While philanthropic donor support was critical to initiating the networks, members will need to mobilize the majority of future resources from their governments and other sources in order to sustain their efforts. Sustaining networks also requires maintaining the interest and support from national governments and future generations of public health leaders, particularly when founding members/partners retire; and continuing engagement and support from other stakeholders and ministries, including customs, border security. Because surveillance is a public good at national, regional and global levels, asking member countries, international organizations, and other partners to invest is a legitimate approach.

In addition to challenges around financing and sustainability of networks, language and cultural differences, along with the broader geopolitical context, often present barriers to effective cooperation. This is true even though regional networks organize in response to shared threats and challenges.

## Conclusions

**Fig. 1:**
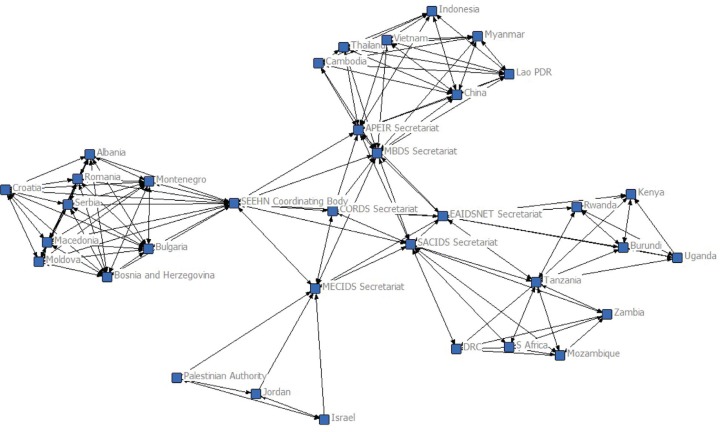
A social network graph illustrating the connections among countries and regional networks in CORDS (CORDS=Connecting Organizations for Regional Disease Surveillance; MBDS=Mekong Basin Disease Surveillance network; EAIDSNet=East Africa Integrated Disease Surveillance Network; SEEHN=Southeastern European (SEE) Health Network; MECIDS=Middle East Consortium for Infectious Disease Surveillance; APEIR=Asia Partnership on Emerging Infectious Diseases Research; SACIDS=Southern African Centre for Infectious Disease Surveillance). Source: Katherine C. Bond.

Table 1 summarizes the major features of the networks described in this Supplement and connected through CORDS. Practitioner networks began by defining and reporting infectious diseases. They then established cross-border reporting mechanisms that prioritized diseases according to how frequently they would be reported. As relationships matured, countries within the networks undertook joint outbreak investigations and other response efforts, and incrementally included sectors beyond health. In recent years, they have applied pandemic preparedness exercises for joint planning and focused increasing attention on the animal-human health interface. Research networks described in this supplement emerged later, with a focus on multi-sectoral collaboration and research on the animal-human health interface. Figure 1 illustrates how the networks connect through overlapping country membership within regional networks and between regional secretariats.

**Table 1:** A summary of activities undertaken by networks in CORDS

Practitioner-driven networks	Countries Involved	Diseases Monitored through Regular Cross-Border Reporting	Illustrative Joint Response of Regional Significance & Sectors involved	Pandemic Preparedness Exercises Undertaken	Work at the Animal-Human Health Interface	Financing
MBDS (1999)	Cambodia, China (Yunnan and Guangxi Provinces), Lao PDR, Myanmar, Thailand, Vietnam	Acute flaccid paralysis, SARS, cholera, H5N1, dengue fever/dengue hemorrhagic fever, typhoid fever, measles, malaria, pneumonia, HIV/AIDS, tuberculosis	SARS, H5N1, cholera Cross-border response teams comprised of health, customs, immigration, and border officials	Six national tabletop simulation exercises; one regional exercise; multiple provincial and cross-border exercises	Core component of strategic plan; field epidemiology training for veterinarians; border investigations involving human and veterinarian sectors	RF, NTI, Google.org,
EAIDSNet (2000)	Burundi, Kenya, Tanzania, Rwanda, Uganda	Acute haemorrhagic fevers, Cholera, Yellow fever, Measles, Plague, Poliomyelits, Bloody diarrhea Cerebro-spinal meningitis, Neonatal tetanus, Rabies, Malaria, Typhoid fever, Diarrhea in <5 years	Cholera, ebola, marburg, wild polio virus. Cross-border response teams with veterinary, human health, security, biosecurity and communication	Regional tabletop simulation exercise; national-level desk top exercises; cross-border field simulation (Kenya-Uganda border)	Component of transboundary integrated disease surveillance efforts; veterinarian sector participates	RF, European Union, EAC
SEEHN (Southeastern European Health network) (2001)	Albania, Bosnia and Herzegovina, Bulgaria, Croatia, Moldova, Montenegro, Romania, former Yugoslav Republic of Macedonia	Influenza, brucellosis, measles, Crimean-Congo hemorrhagic fever, West Nile Fever, salmonella; vaccine preventable diseases	Works with experts from WHO, European CDC, UK Department of Health. Involvement of health and other sectors (veterinary, customs, ecology, wildlife, etc)	Joint preparation of influenza pandemic preparedness plans at both national and regional levels; introduction of molecular techniques into influenza surveillance laboratories region-wide. Regional table top pandemic preparedness exercise	Broader investigation of Brucellosis and pandemic preparedness with involvement of veterinary sector.	SEE countries governments Greek government French government Belgian government
MECIDS (2003)	Kingdom of Jordan, Israel, the Palestinian Authority	Salmonella, shigella, H5N1, leishmaniasis	Foodborne disease, H5N1, H1N1 Ministries of human and animal health and agriculture	Tabletop simulation and semi-functional drills; trainings, data sharing	Training and exercises conducted across sectors for identification and response	NTI; World Bank
Research-driven networks	Countries Involved	Types of Research	Sectors Involved	Pandemic Preparedness Exercises	Animal-Human Health Interface	Financing

APEIR (2006)	Cambodia, China, Indonesia, Laos, Thailand and Vietnam	Knowledge generation, policy research, research capacity building, on avian influenza and infectious diseases	Research partnerships with Agriculture, Health, Education, Sciences	n/a	Multi-country research partnerships; Eco-health concepts; e.g., Surveillance and Monitoring of Avian Influenza in Migratory Birds; Policy impact assessments of poultry vaccination	IDRC, Health Systems Research Institute (coordinating office)
SACIDS (2008)	Democratic Republic of Congo, Mozambique, South Africa, Tanzania, Zambia	Research using geo-spatial analyses, resource mapping and preparedness analyses, research on specific diseases, applications of mobile technology	Research partnerships with human, livestock and wildlife health	n/a	Climate dependent vector-borne diseases; diseases with potential inter-species concern; disease of economic and food security importance; bacterial rare diseases; dangerous emerging diseases	RF, Google.org, NTI, Wellcome Trust

Two key factors contributed to the emergence and growth of regional infectious disease surveillance networks. First and foremost, the ongoing engagement of governments, coupled with the longevity of membership of individuals in the networks, has enabled some networks to extend over more than a decade and all networks to build incrementally on knowledge and experience. In many of these networks, senior members have actively mentored the younger generation to take on increasing leadership and decision-making roles. Second, the networks have leveraged multiple initiatives and have adapted their respective governance structures to the context of their regional institutional landscapes. For example, EAIDSNet was able to integrate into the EAC after the EAC became a treaty organization, while MBDS remained an informal network of governmental and private philanthropic and technical partners until it was formalized as a nationally registered foundation in 2012. MBDS was also the basis for the formation of the ACMECS, ASEAN+3FETN and APEIR. As the networks become institutionalized, they will face a new set of challenges, from identifying diversified and sustainable sources of funding to adapting to newly emerging public health threats. Given that the drivers of emerging infectious diseases are likely to continue or increase, we expect to see ongoing interest in this approach and the establishment of new networks in other regions, such as South Asia. The last 15 years has demonstrated the strong likelihood they will succeed.
